# Modeling the hepatitis A epidemiological transition in Brazil and Mexico

**DOI:** 10.1080/21645515.2017.1323158

**Published:** 2017-05-08

**Authors:** Thierry Van Effelterre, Adrienne Guignard, Cinzia Marano, Rosalba Rojas, Kathryn H. Jacobsen

**Affiliations:** a GSK Vaccines, Wavre, Belgium; b Centro de Investigaciones en Salud Poblacional, Instituto Nacional de Salud Pùblica, Morelos, México; c Department of Global and Community Health, George Mason University, Fairfax, VA, USA

**Keywords:** clean water, hepatitis A, Latin America, mathematical model, seroprevalence, urbanization

## Abstract

*Background*: Many low- to middle-income countries have completed or are in the process of transitioning from high or intermediate to low endemicity for hepatitis A virus (HAV). Because the risk of severe hepatitis A disease increases with age at infection, decreased incidence that leaves older children and adults susceptible to HAV infection may actually increase the population-level burden of disease from HAV. Mathematical models can be helpful for projecting future epidemiological profiles for HAV.

*Methods*: An age-specific deterministic, dynamic compartmental transmission model with stratification by setting (rural versus urban) was calibrated with country-specific data on demography, urbanization, and seroprevalence of anti-HAV antibodies. HAV transmission was modeled as a function of setting-specific access to safe water. The model was then used to project various HAV-related epidemiological outcomes in Brazil and in Mexico from 1950 to 2050.

*Results*: The projected epidemiological outcomes were qualitatively similar in the 2 countries. The age at the midpoint of population immunity (AMPI) increased considerably and the mean age of symptomatic HAV cases shifted from childhood to early adulthood. The projected overall incidence rate of HAV infections decreased by about two thirds as safe water access improved. However, the incidence rate of symptomatic HAV infections remained roughly the same over the projection period. The incidence rates of HAV infections (all and symptomatic alone) were projected to become similar in rural and urban settings in the next decades.

*Conclusion*: This model featuring population age structure, urbanization and access to safe water as key contributors to the epidemiological transition for HAV was previously validated with data from Thailand and fits equally well with data from Latin American countries. Assuming no introduction of a vaccination program over the projection period, both Brazil and Mexico were projected to experience a continued decrease in HAV incidence rates without any substantial decrease in the incidence rates of symptomatic HAV infections.

## Introduction

Hepatitis A disease is an acute inflammation of the liver caused by the hepatitis A virus (HAV). It is transmitted mainly by the fecal-oral route following person-to-person contacts but may also be transmitted by contaminated food or water.^1^ The incidence of HAV infection is associated with inadequate sanitation and limited access to safe water and improvements of sanitation and safe water supply reduce the risk of infection.^1-3^

The risk and severity of symptomatic HAV infection is directly related to age. Young children usually have no or few symptoms, whereas most older children and 80–90% of adults develop an icteric infection (jaundice).^1,3,4^ HAV infection induces lifelong immunity after recovery.^1^ In regions with high incidence rates, nearly all children become infected early in life, so most cases are asymptomatic and overt outbreaks are infrequent.^1,5^ In regions experiencing decreasing HAV incidence rates after improvements in sanitation and water access, the mean age at HAV infection usually increases and outbreaks become more common.^1,6^ The higher mean age at HAV infection may potentially lead to an increased incidence of symptomatic HAV infections, so a shift from intermediate or high towards low endemicity may actually lead to an increased burden of disease. WHO recommends that vaccination against HAV should be integrated into the national vaccination schedule for children aged ≥ 1 y if there is a change in the endemicity of hepatitis A from high to intermediate.^7^

Incidence data for HAV infections are very incomplete, because nearly all the cases in regions with high endemicity occur in young children and even symptomatic cases are often underreported.^8,9^ Therefore, the best option is to use age-specific seroprevalence data to back-calculate incidence rates over time. Seroprevalence studies test blood for anti-HAV IgG or IgG/IgM antibodies, where IgM is a marker of acute or recent infection and IgG a marker of past infection.^10^ Plots of age-stratified seroprevalence curves based on cross-sectional serosurveys reveal information about HAV endemicity at the time of data collection and to some extent also suggest how HAV incidence has developed over time.^1^

The seroprevalence of HAV varies over geographic and socioeconomic regions. Many low- and middle-income countries had high HAV endemicity levels but are in the process of transitioning to low endemicity.^1,6^ This shift has been observed in both Brazil and Mexico^11-25^ 2 countries we selected for this study because of seroprevalence data availability for both rural and urban study populations.

**Figure 1.**
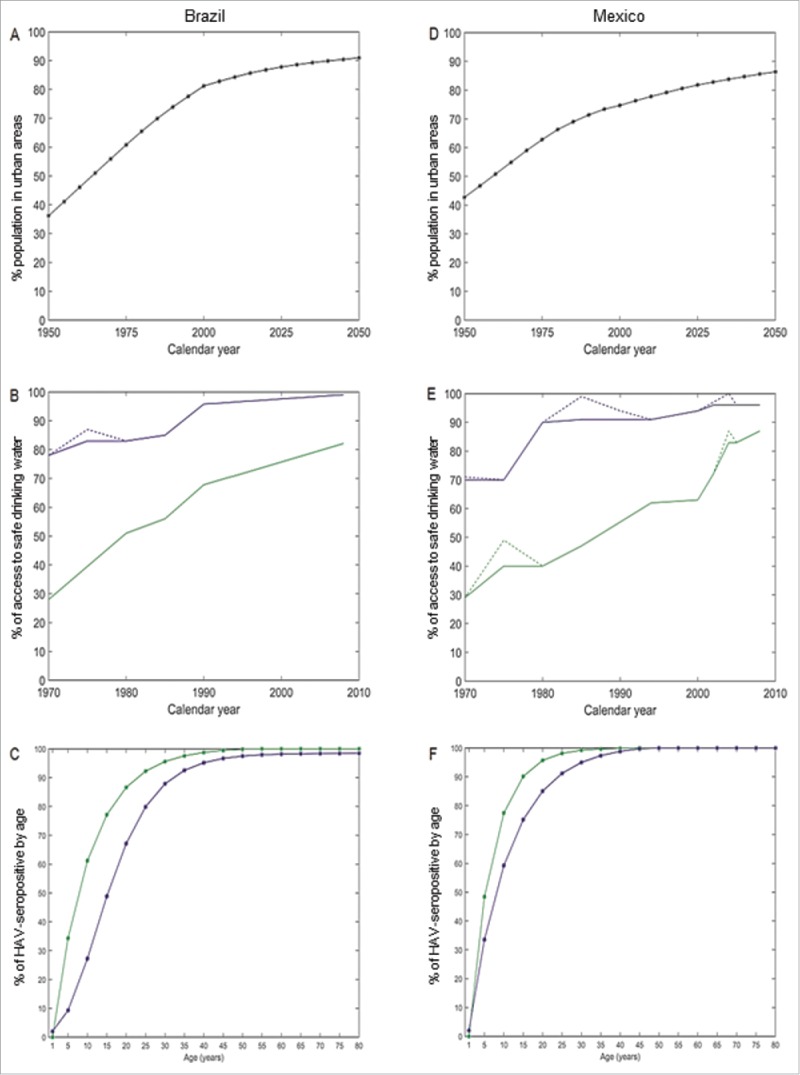
Urbanization, access to safe drinking water and seroprevalence of anti-HAV antibodies. (A) Percentage of the total population in urban areas over time in Brazil; (B) Percentage with access to safe drinking water over time in Brazil; (C) Percentage of HAV-seropositive by age (synthesized seroprevalence curves by setting) in Brazil. (D) Percentage of the total population in urban areas over time in Mexico; (E) Percentage with access to safe drinking water over time in Mexico; (F) Percentage of HAV-seropositive by age (synthesized seroprevalence curves by setting) in Mexico. For A and D black curves: by 5 calendar years. For B-C-E-F, green: rural setting; blue: urban setting; dashed lines: observed data; continuous lines: adjusted data (taking into account stricter definitions of access to clean drinking water and assuming the actual access never declines).

Our objective was to project the epidemiology of HAV in Brazil and Mexico up to 2050, based on demography, urbanization, access to safe water and HAV seroprevalence data (Fig. 1, see additional information in the Materials and Methods section). For this purpose we used a mathematical epidemiological model. We present the results for each country separately, since the aim of this modeling exercise was to examine the applicability of this HAV epidemiological model to 2 Latin American countries. While it is appropriate to compare the broad trends of the country projections, the purpose was not to compare the model outcomes between the 2 countries quantitatively.

## Results

### Brazil

**Figure 2.**
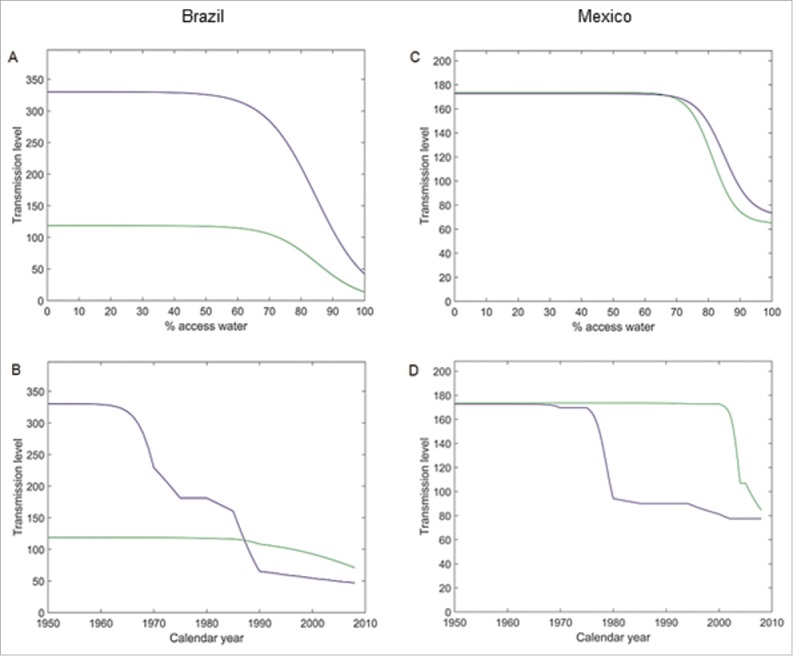
Transmission level vs access to safe drinking water and transmission level over time. (A) Transmission level vs percentage with access to safe drinking water in Brazil; (B) Derived transmission level over time in Brazil; (C) Transmission level vs percentage with access to safe drinking water in Mexico; (D) Derived transmission level over time in Mexico. Green: rural setting; blue: urban setting.

When access to safe water was 100% vs. 0%, the model projected an 8.7-fold and 7.8-fold decrease of the transmission level in rural and urban settings, respectively (Fig. 2A) and a consistently higher transmission level in urban settings. The derived transmission level over time was higher in urban settings until the late 1980s and became higher in rural settings afterwards (Fig. 2B).

**Figure 3.**
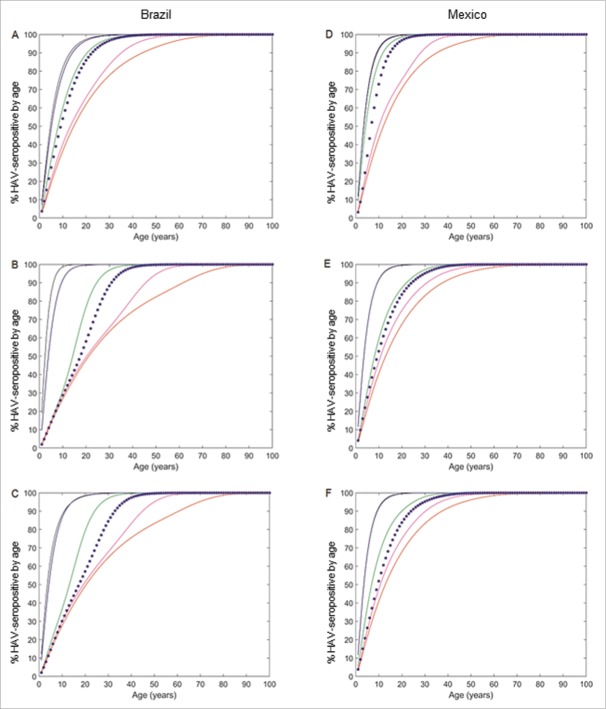
Model-projected anti-HAV IgG seropositive in the different settings. Model-projected anti-HAV IgG seropositive in rural setting (A), urban setting (B) and at country level (C) in Brazil and rural setting (D), urban setting (E) and at country level (F) in Mexico every 25 calendar years between 1950 and 2050. Black: 1950; blue: 1975; green: 2000; magenta: 2025; red: 2050. Blue stars in rural and urban settings: years of the seroprevalence surveys (Table S1); at country level: 2010 as reference year.

**Figure 4.**
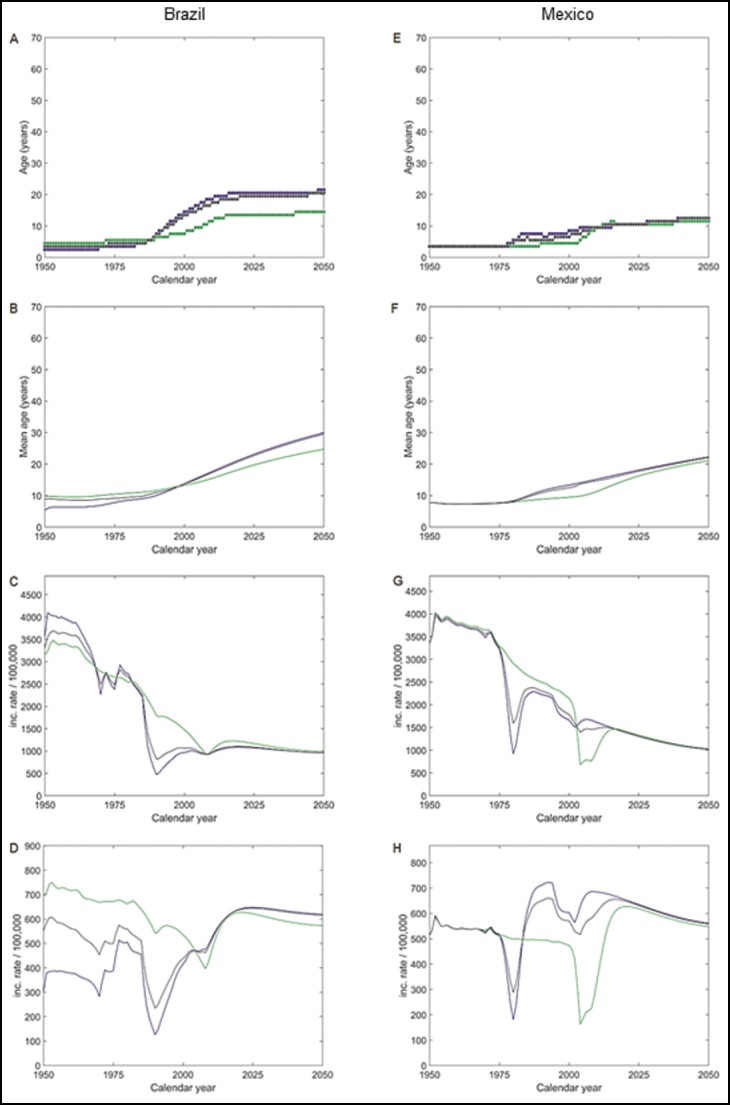
Projected HAV epidemiological outcomes over time from 1950 to 2050. (A) Projected first age at which at least 50% of the population is anti-HAV seropositive (AMPI) in Brazil; (B) Projected mean age of symptomatic HAV-infection in Brazil; (C) Projected annual incidence rate of all HAV-infections (per 100,000) in Brazil; (D) Projected annual incidence rate of symptomatic HAV-infections (per 100,000) in Brazil. (E) Projected first age at which at least 50% of the population is anti-HAV seropositive (AMPI) in Mexico; (F) Projected mean age of symptomatic HAV-infection in Mexico; (G) Projected annual incidence rate of all HAV-infections (per 100,000) in Mexico; (H) Projected annual incidence rate of symptomatic HAV-infections (per 100,000) in Mexico. Green: rural setting; blue: urban setting; black: country level.

The projected age-seroprevalence curves are shown in Figs. 3A–3C and S3A-B. The projected AMPI was consistently higher in urban than in rural settings from 2000 onwards and increased progressively everywhere between 1975 and 2025 and then plateaued for the subsequent 25 y (Fig. 4A and Table 1). The projected mean age of symptomatic HAV-infection increased progressively over time in both settings, especially in urban settings where it was lower than in rural settings for several years after 1950. The projected mean age of symptomatic HAV-infection in 2050 was 25 and 30 y in rural and urban settings, respectively (Fig. 4B and Table 1).

**Table 1.** Epidemiological outcome projections from the model over time.

		Year				
Outcome	Setting	1950	1975	2000	2025	2050
Brazil						
First age with at least 50% HAV-positive (AMPI)	Rural	4	5	7	13	14
	Urban	2	3	14	20	21
	Country	3	4	13	19	20
Mean age of symptomatic HAV infection	Rural	10	10	13	20	25
	Urban	5	8	14	23	30
	Country	9	9	14	23	29
Incidence rate of all HAV infections (per 100,000/year)	Rural	3163	2652	1477	1165	983
	Urban	3566	2381	968	1072	955
	Country	3309	2487	1063	1083	958
Incidence rate of symptomatic HAV infections (per 100,000/year)	Rural	695	672	535	622	573
	Urban	303	388	416	647	620
	Country	553	499	439	644	615
Mexico						
First age with at least 50% HAV-positive (AMPI)	Rural	3	3	4	10	11
	Urban	3	3	8	10	12
	Country	3	3	6	10	12
Mean age of symptomatic HAV infection	Rural	8	8	9	16	21
	Urban	8	8	13	18	22
	Country	8	8	13	18	22
Incidence rate of all HAV infections (per 100,000/year)	Rural	3348	3292	2143	1332	1027
	Urban	3346	3234	1665	1305	1017
	Country	3347	3256	1786	1310	1019
Incidence rate of symptomatic HAV infections (per 100,000/year)	Rural	513	511	474	618	547
	Urban	515	518	603	634	561
	Country	514	516	571	631	559
AMPI = Age at the midpoint population immunity						

**Figure 5.**
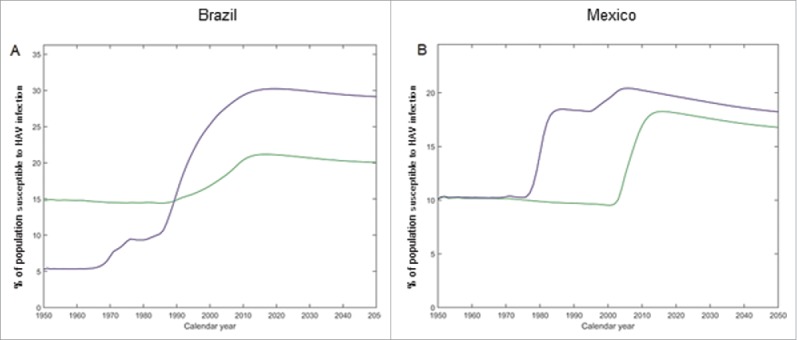
Projected percentage of population susceptible to HAV infection. Projected percentage of population (all age groups) susceptible to HAV infection from 1950 to 2050, in Brazil (A) and in Mexico (B). Green: rural setting; blue: urban setting.

The projected incidence rates of all HAV infections decreased considerably in both settings until about 2000, leveling off thereafter to a setting-independent annual incidence rate of around 1000 cases per 100,000 individuals from about 2010 and onwards (Fig. 4C and Table 1). The transitory re-increase of the incidence rate in urban settings from around the late 1980s until 2010 may be explained by replenishment of the number of susceptible (see Fig. 5A) following the sharp decrease in the number of infections after urban safe water access improved from about 80% in 1980 to almost 100% in 2008 (Fig. 1B).

The incidence rate of symptomatic HAV infection projected for rural settings showed a minor decrease over the entire projection period, whereas its urban counterpart doubled. By 2050, the projected incidence rate of symptomatic HAV infections was around 600 cases per 100,000 individuals in both settings (Fig. 4D and Table 1).

Constraining 4 rather than 3 parameters of the transmission model in the sensitivity analysis had little impact on the projected outcomes for 2025 and 2050. At intermediate time points, the pattern observed was that constraining 4 parameters led to a clear decrease of the incidence of symptomatic HAV infections in rural and a large increase in urban settings in 1975 and 2000 (Table S4A).

### Mexico

When access to safe water was 100% vs. 0%, the model projected a 2.7 and 2.4-fold decrease of the transmission level in rural and urban settings, respectively (Fig. 2C). The derived transmission level over time was similar in both settings until around 1975. Then, a sharp decrease in the transmission level occurred in urban settings followed by a similar decrease in rural settings 30 y later (Fig. 2D).

The projected age-seroprevalence curves are shown in Figs. 3D–3F and S3C-D. The projected AMPI was stable and similar in both settings until 1975, more than doubling in urban but remaining stable in rural settings from 1975 to 2000, followed by an increase everywhere; by 2025 the projected AMPIs were similar in both settings (Fig. 4E and Table 1). The mean age of symptomatic HAV cases was constant and similar in both settings until 1975, when it started to increase, most pronounced in urban settings until 2000 but after that a faster increase occurred in rural settings with the result that the projected mean age of symptomatic HAV infection in 2050 was around the early 20s in both settings (Fig. 4F and Table 1).

In both settings, the projected incidence rate of all HAV infections decreased by more than two thirds over the projection period. The decrease in incidence occurred earlier in urban settings, with incidence falling by 50% from 1975 to 2000, but slowing down thereafter whereas the decrease in rural settings was more gradual (Fig. 4G and Table 1). In both settings, a transitory re-increase in the HAV incidence rate was projected, occurring 30 y earlier in urban than in rural settings and related to replenishment of susceptible individuals (Fig. 5B).

The projected incidence rates of symptomatic HAV infections were similar in both settings at the beginning and at the end of the period with fluctuations in between and a modest increase overall (Fig. 4H and Table 1).

Constraining 4 rather than 3 parameters of the transmission model in the sensitivity analysis had relatively little impact on the projected outcomes for 2025 and 2050. At the earlier time points, constraining 4 parameters led to a clearly higher incidence rate of symptomatic HAV infections in both settings (Table S4B).

## Discussion

The model worked well for both countries, as it could be calibrated with a good fit to the country- and setting-specific synthesized seroprevalence curves estimated based on observed age- and setting-specific seroprevalence data. The model-projected epidemiological outcomes are qualitatively broadly similar for the 2 countries and first of all characterized by a decrease in endemicity and increased AMPI both in rural and urban settings as a consequence of improved access to safe water, partly as a result of growing urbanization.

Secondly, the marked overall decrease in the incidence of HAV infections was not accompanied by a corresponding fall in the incidence of symptomatic HAV infections. On the contrary, the incidence in absolute terms of symptomatic HAV infections remained stable or increased somewhat leading, obviously, to a rise in symptomatic HAV infections as a proportion of all HAV disease.

Thirdly, the sharp decrease in the incidence of all HAV infections following closely after marked improvements in the access to safe water reaching more or less all of the population in both settings was superseded by a transitory re-increase in the incidence before returning to a continued decrease. Our tentative explanation of this projected phenomenon was that the sharply falling HAV infection incidence led to replenishment in the number of susceptible individuals.

Among the strengths of the model is that it is dynamic in terms of both demographics and epidemiology and stratified by age and by setting. Thereby it takes into account changing demographics (including urbanization) and changes in the age-specific risk of symptomatic HAV infection. The subpopulations in the 2 settings are dynamically coupled over time by migration from rural to urban areas, which accounts for a substantial portion of the decreased incidence rates until around 2000. The projections indicate that the incidence of all HAV infections and of symptomatic cases alone will be independent of setting from the present until 2050.

Applying this epidemiological HAV model requires access to observational data on seroprevalence of anti-HAV antibodies stratified not only by age but also by rural vs. urban settings. Such data are not available for many low- and middle-income countries. However, many of the other input data required, such as demographics, urbanization and access to safe water are available.^26-28^ A next step for exploring the usefulness of the model might be to see how well it projects epidemiological outcomes based solely on those data. Extrapolation may potentially be considered but only for broad trends, because more precise extrapolations require that the relation between HAV transmission and water access be similar. This, however, does not appear to be the case, since the transmission factor function, which characterizes the decrease of HAV transmission as a function of improved access to safe water, differs between countries and settings (Fig. S5). The proportion of the population with access to safe water at which the transmission factor function started to decrease was approximately 40% in Brazil and 65% in Mexico and with full access the value of the transmission factor function was approximately 0.15 in Brazil but 0.40 in Mexico.

The limitations of the model, which require cautious interpretation of the projections, include the assumptions that the age distribution is identical in rural and urban areas and that rural-to-urban migration is independent of age. Furthermore, it was assumed that 10% of the FOI in the base year was caused by other than person-to-person transmission, because the correct value cannot be measured empirically. Moreover, the observational data on age- and setting-specific seroprevalence used to estimate the parametric seroprevalence curves for model calibration are limited and of uncertain and unequal quality.

Access to clean water was fixed at the level of 2008, the year of the most recent data available. In both countries, access to clean water was almost at 100% in urban areas, leaving very little room for improvement. In rural areas, clean water access was already at 80% or higher in both countries. The remaining rural areas without improved water access are generally in very remote places, where the improvements will likely be slower than in more accessible places and we did not want to overestimate the rate of improvement.

Finally, the model does not incorporate any estimate of the impact of vaccination on HAV trends. Since both countries recently included hepatitis A vaccination in their pediatric immunization schedules, their burden from HAV disease will likely develop differently from the model-projected outcomes. The introduction of universal mass vaccination for HAV infection in countries with intermediate endemicity has led to a marked decrease in the incidence of hepatitis A in both the vaccinated and non-vaccinated age groups.^29^ However, the model projections do demonstrate that reliable access to clean water in both rural and urban areas is a significant predictor of the HAV epidemiological transition.

## Conclusions

The HAV model worked well for both Latin American countries with good fits between the model projections and the observational data for the time points when seroprevalence surveys were carried out. These good fits make us confident that the model projections for the near future are reasonably accurate in the absence of vaccination. Extrapolation of the model-projections of epidemiological outcomes to other countries undergoing the HAV epidemiological transition but without recently collected detailed HAV seroprevalence data might be of interest and useful. However, if countries differ with regard to the relationship between improvements in access to clean water and HAV transmission, which seems to be the case for the 3 countries to which the model has been applied so far (Brazil, Mexico and Thailand), such extrapolations become more problematical and uncertain. Applying the HAV model to other countries with detailed HAV seroprevalence data available may provide better insight into the factors underlying the differences in the relationship between safe water access and HAV transmission.

## Materials and methods

The epidemiological HAV model has been described at length in a previous paper^30^ with additional details provided in the supplement to that manuscript. Here, we give a brief summary of the modeling process and the data inputs.

### Modeling key steps and data sources

The sources of the data used as model inputs are listed in Table S1 in the Supplementary Material (SM). The modeling included the following key steps.

1) The parameters of the demographic model were estimated by calibrating it to United Nations data and projections about total population sizes by age and level of urbanization (Figs. 1A/D and S1,^26,27^). The demographic model is dynamic, based on observations and projected data for births, level of urbanization and age-specific mortality.
2) For each country and for rural and urban settings separately, parametric models were fitted to the observed age-specific seroprevalence data to obtain cross-sectional, setting-specific parametric seroprevalence curves (Figs. 1C/F and S2). These parametric seroprevalence curves were used to estimate the parameters of the transmission model (described in point (3)). The parameters were estimated by calibrating the model-projected seroprevalence curves to the parametric seroprevalence curves. This calibration was done by minimizing the sum of squares between the setting-specific model-projected and parametric seroprevalence curves allowing the model-projected seroprevalence curves to be as close as possible to the respective seroprevalence curves based on observed data.
3) For each setting separately, the HAV transmission was modeled as a decreasing function of the setting-specific percentage of the population with access to safe water. Data about safe water (Fig. 1B/E) were adjusted to ensure access increased monotonically over time, and a linear increase in access was assumed for 1950–1970 and estimated by calibration (Table S3). For the future projections, the percentage of individuals with access to safe water was fixed at the percentage observed at the latest time point with data available (2008).^28^
4) The model was used to project epidemiological outcomes from 1950 to 2050, including: (i) the age at midpoint of population immunity (AMPI, the first age at which at least 50% of the population is HAV seropositive^27^); (ii) the mean age of cases of symptomatic HAV infection; and (iii) the incidence rates of HAV infections, both all and symptomatic only.

All numerical simulations were performed using *Matlab* R2013a (The MathWorks Inc., Natick, MA, USA).

### Dynamic transmission model of HAV natural history

The transmission model is constructed as a deterministic, compartmental, age-stratified, dynamic transmission model for the natural history of HAV with 5 distinct infection states: protected by maternal antibodies (assumed mean duration 9 months); susceptible to HAV infection; infected by HAV but not infectious (2 weeks); infectious (3 weeks); recovered and immune (assumed lifelong immunity following infection).

The model was stratified by age (one-year age groups) and by setting (rural and urban). The age-specific proportion of HAV infections assumed symptomatic were based on a disease model (Fig. S4^4^). Person-contacts were assumed to occur within each separate setting, so the only interaction between settings is the rural-to-urban migration.

The models used in the present study differ on certain points from the model described in detail previously^30^:

• The demographic data were pre-processed to smooth the urbanization rate and number of births over time.
• Emigration from the country was included for Mexico, because of its importance for national demographic changes.
• In the previous version, all newborns were assumed protected by maternal antibodies. In the adapted models used here, some newborns were assumed susceptible at birth, with the fraction of susceptible newborns determined by the time-specific distribution of the age of mothers of newborns and the time-, age- and setting-specific proportion of HAV-seropositive in the population.

### Force of infection

The force of infection (FOI), i.e., the rate at which susceptible individuals become infected, was determined as the sum of the risk of person-to-person transmission and the risk from other causes, like food- or water-borne transmissions and importation of HAV. The risk of person-to-person transmission was modeled as depending in a multiplicative way on 3 setting-specific factors: (i) the percentage of infectious individuals; (ii) a transmission parameter, β, accounting for contacts between individuals and the risk per contact; and (iii) the decrease in transmission over time related to the proportion of the population with safe water access. The transmission level (with the dimension 1/time, see Fig. 2) was derived as the product of the transmission parameter β and a country- and setting-specific transmission factor defined as a function of the calendar year.

For each setting, a function was defined to characterize the decrease of HAV transmissions related to improved access to safe water. This relation was modeled as a sigmoidal function decreasing monotonically as a function of the setting-specific percentage of the population with access to clean water. The risk of a susceptible individual to become infected by other causes than person-to-person transmission was fixed at 10% of the FOI prior to 1950 (with demography and HAV epidemiology assumed to be in steady-state prior to 1950). The same decreasing factor as a function of safe water access was applied to transmission from other causes than person-to-person transmission.

### Base case and sensitivity analyses

For the base case, the relative difference between rural and urban settings for 3 of the transmission model parameters was constrained to be at most 10%. These 3 parameters are those characterizing the sigmoidal function decreasing monotonically as a function of the access to safe water (α, δ and f (Table S3)). In sensitivity analyses, the model was calibrated and the outcomes projected when constraining 4 of the transmission model parameters, i.e., the transmission parameter β in addition to the 3 mentioned above (Table S3). The projected epidemiological outcomes for the base case are presented in the results section and of the sensitivity analyses in the SM.

## Supplementary Material

Supplemental_Material.docx

## Abbreviations

AMPI: age at the midpoint of population immunity
FOI: Force of infection
HAV: hepatitis A virus
