# Supplementary material for: Modeling the hepatitis A epidemiological transition in Brazil and Mexico
Source: Hum Vaccin Immunother. 2017 May 8;13(8):1942–51. doi: 10.1080/21645515.2017.1323158 (PMC5557237; doi:10.1080/21645515.2017.1323158)
Supplement: Supplemental_Material.docx [file khvi-13-08-1323158-s001.docx]

**Supplementary material**

**Table S1. Data sources**

| **Country** | **Type** | **Details and source** |
| --- | --- | --- |
|  | Demographic | Population size over time (stratified by 5-year age groups, by 5 calendar years periods) [[2](#_ENREF_28)7] |
|  |  | Mortality with age-specific death rates [[2](#_ENREF_28)7,28] |
|  |  | Country-specific percentages of urban and rural population [[2](#_ENREF_26)6] |
| Brazil |  | Number of newborns by age of the mother (United Nations estimates/projections until 2100 (2014 update) [27] |
|  | Epidemiological | In rural setting: Vitral et al. [15]  In urban setting: Ximenes et al. [10] |
|  | Access to safe drinking water | Percentage of the population with access to clean drinking water over 1970-2008 [[26](#_ENREF_27)]; data were adjusted to ensure access increased monotonically over time, and a linear increase in access was assumed for 1950-1970 due to lack of data and estimated by the model |
|  | Demographic | Population size over time (stratified by 5-year age groups, by 5 calendar years periods) [[2](#_ENREF_28)7] |
|  |  | Mortality with age-specific death rates [[2](#_ENREF_28)7,31] |
|  |  | Country-specific percentages of urban and rural population [[2](#_ENREF_26)6] |
|  |  | Migration rates: United Nations data [27] |
| Mexico |  | Number of newborns by age of the mother (United Nations estimates/projections until 2100 (2014 update) [27] |
|  | Epidemiological | Data by setting from ENSANUT 2006. Kindly provided by the Instituto Nacional de Salud Publica in Mexico for the purpose of populating the model |
|  | Access to safe drinking water | Percentage of the population with access to clean drinking water over 1970-2008 [[2](#_ENREF_27)6]; data were adjusted to ensure access increased monotonically over time, and a linear increase in access was assumed for 1950-1970 due to lack of data and estimated by the model |

The reference numbers refer to the list of references of the core manuscript

**A. The demographic model**

For additional information, see the Supplementary Materials in [23].

**Table S2: Estimates of the parameters for the demographic model through calibration**

| **Country** | **Parameter** | **Estimate** |
| --- | --- | --- |
|  | Growth rate at steady state (prior to 1950) | 0.0279 |
|  | Rate of exponential decrease of the age-specific death rates in <70-year olds after 2010 as compared to death rates from 2012 [28] | 0.0206 |
| Brazil | Rate of exponential decrease of the age-specific death rates in 70+ years old after 2010 as compared to death rates from 2012 [28] | 0.0115 |
|  | Annual migration rates from rural to urban setting (calendar year-specific) | Range: 0 - 0.0381  Mean: 0.0132  Median: 0.0148 |
|  | Growth rate at steady state (prior to 1950) | 0.0274 |
|  | Rate of exponential decrease of the age-specific death rates in <70-year olds after 2010 as compared to death rates from 2012 [28] | 0.0109 |
| Mexico | Rate of exponential decrease of the age-specific death rates in 70+ years old after 2010 as compared to death rates from 2012 [28] | 0.0118 |
|  | Annual migration rates from rural to urban setting (calendar year-specific) | Range: 0 - 0.0209  Mean: 0.0097  Median: 0.0122 |

The reference numbers refer to the list of references of the core manuscript

**Fig. S1 Total population over time**

| **A** | **B** |
| --- | --- |
| **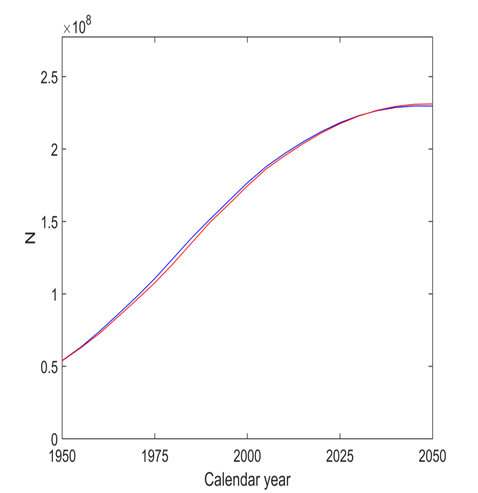** | **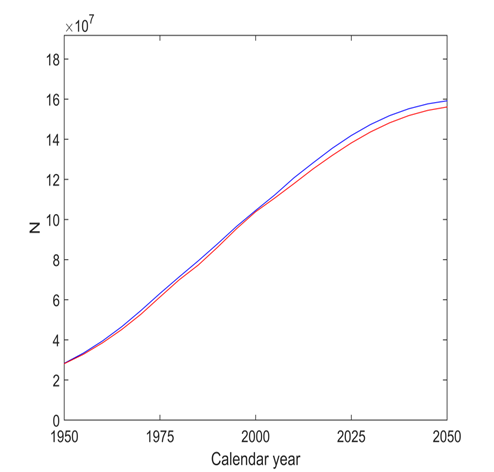** |

Blue: Model-based projections; Red: Observed data and projections from the UN

Figure S1 shows the total population over time projected by the model versus the UN data/projections. A: Brazil, B: Mexico

**B. Seroprevalence curves**

For additional information, see the Supplementary Materials in [23].

**Fig. S2 Estimated synthesized seroprevalence curves**

| **A** | **C** |
| --- | --- |
| **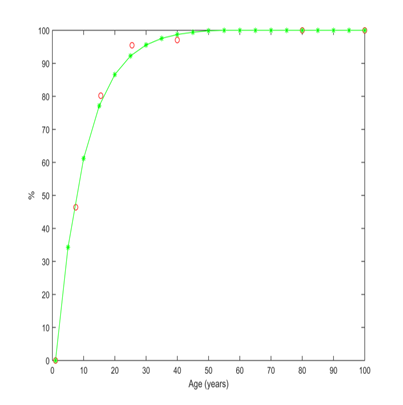** | 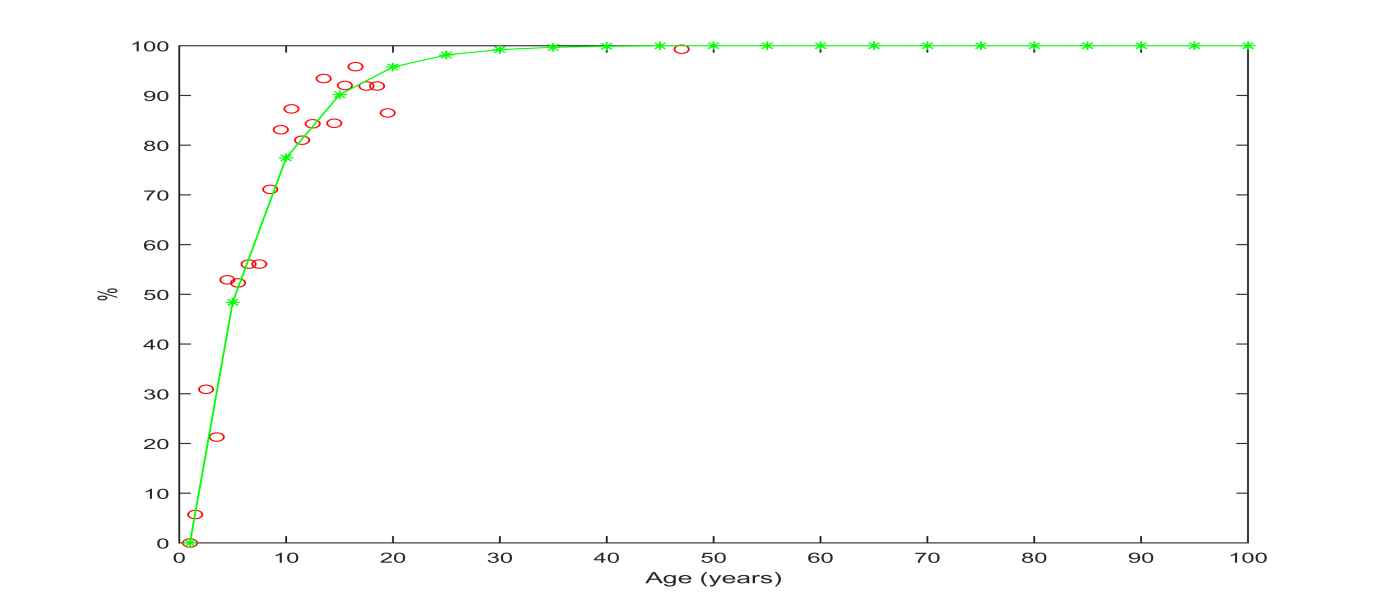 |
| **B** | **D** |
| 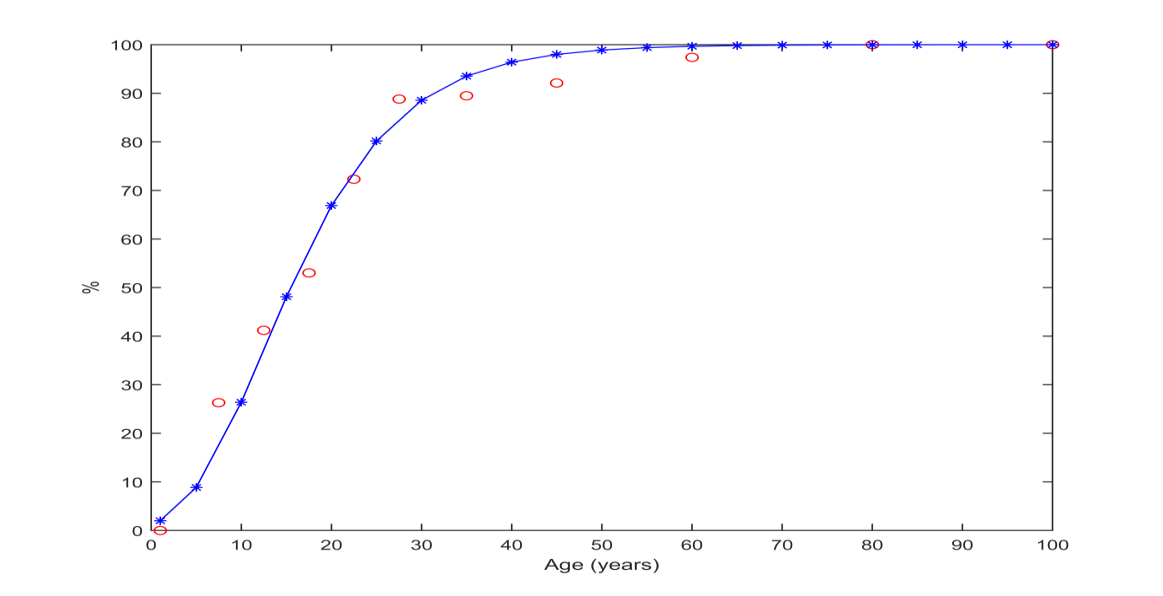 | **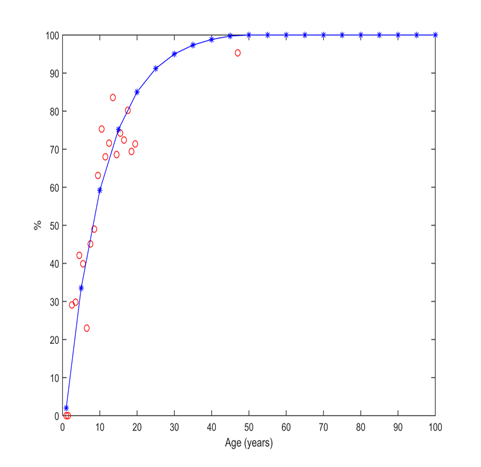** |

Red dots: Data points from the seroprevalence data set(s) (Reference, see Table S1)

Green curves: Parametric curves fit to the seroprevalence data sets, rural settings

Blue curves: Parametric curves fit to the seroprevalence data sets, urban settings

(A) Brazil, rural setting; (B) Brazil, urban setting; (C) Mexico, rural setting; (D) Mexico, urban setting

**Fig. S3 Percentage anti-HAV positive per age group and setting. Model projections versus synthesized seroprevalence curves**

| **A** | **C** |
| --- | --- |
| **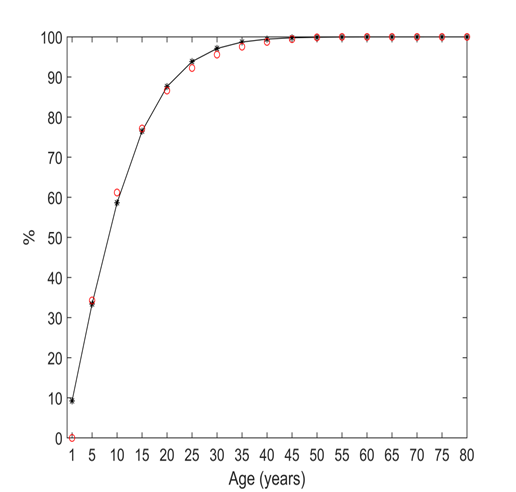** | **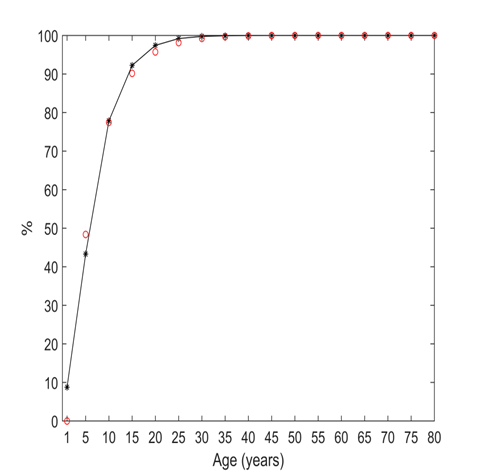** |
| **B** | **D** |
| **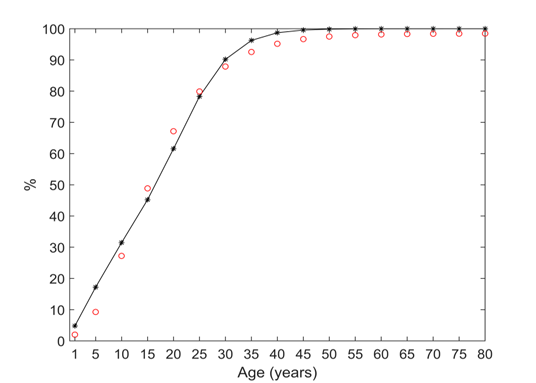** | **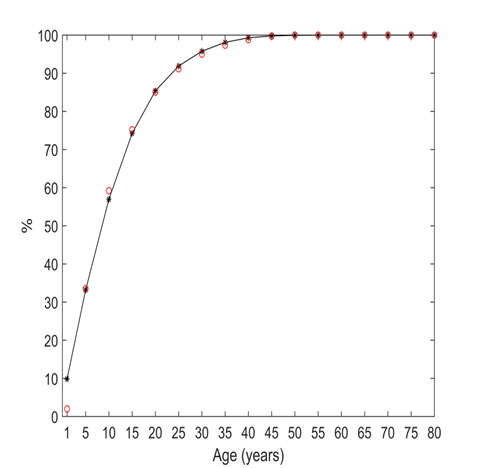** |

Red circles: Estimated synthesized seroprevalence data by age group

Black stars: Model projections at the same age points as in the synthesized seroprevalence data

Black curves: Model-based seroprevalence curves (years of observed data, see Table S1)

(A) Brazil, rural setting; (B) Brazil, urban setting; (C) Mexico, rural setting; (D) Mexico, urban setting

**C. Access to clean drinking water**

For the explanation of the assumptions made for access to safe drinking water, see the Supplementary Materials in [23].

**D. Percentage of symptomatic icteric HAV infection by age (model by Armstrong and Bell [4])**

**Fig. S4 Percentage of HAV infections that are icteric (model by Armstrong and Bell)**

| **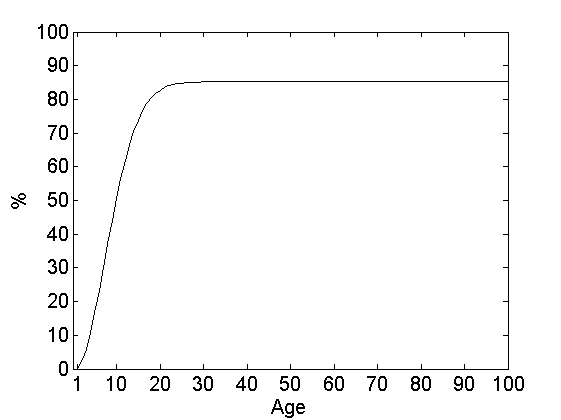** |
| --- |

**E. Estimation of the parameters of the transmission model through calibration**

For the explanation of the estimation by minimization of the sum of squares (SSQ), see the Supplementary Materials in [23].

**Table S3: Estimated parameters for the transmission model for the base case**

| **Country** | **Parameter** | **Setting** | Parameter estimate |
| --- | --- | --- | --- |
|  | **β** | **Rural** | 118.4523 |
|  |  | **Urban** | 330.3654 |
|  | **α** | **Rural** | 51.4644 |
|  |  | **Urban** | 47.7593 |
| **Brazil** | **δ** | **Rural** | 33.5672 |
|  |  | **Urban** | 36.7795 |
|  | **f** | **Rural** | 949.2504 |
|  |  | **Urban** | 1008.1 |
|  | **p_1950_** | **Rural** | 3.8977*10^-12 |
|  |  | **Urban** | 5.7125*10^-8 |
|  | Total SSQ = 299.7712 | | |
|  | **β** | **Rural** | 173.6127 |
|  |  | **Urban** | 172.6250 |
|  | **α** | **Rural** | 65.4990 |
|  |  | **Urban** | 67.5193 |
| **Mexico** | **δ** | **Rural** | 15.7830 |
|  |  | **Urban** | 17.3610 |
|  | **f** | **Rural** | 2.6909 |
|  |  | **Urban** | 2.4468 |
|  | **p_1950_** | **Rural** | 0.0128 |
|  |  | **Urban** | 1.4752*10^-5 |
|  | Total SSQ = 180.8318 | | |

β = Setting-specific transmission parameter accounting for the contacts between individuals and the per-contact risk of HAV transmission

α = Percentage of individuals with access to safe drinking water at which the function F characterizing the decrease of HAV transmission as a function of the percentage of the population having access to safe drinking water starts to decrease from the maximal value of 1

δ = Percentage of individuals with access to safe drinking water at which the function F has its inflexion point starting from α

f = maximal fold decrease of F from its maximum to its minimum value

p_1950_ = The proportion between the percentage of the population with access to safe drinking water in 1950 and 1970, respectively

**F. Factors for transmission**

**Fig. S5 Factor for transmission as a function of water access and derived factor for transmission as a function of time**

| **A** | **C** |
| --- | --- |
| 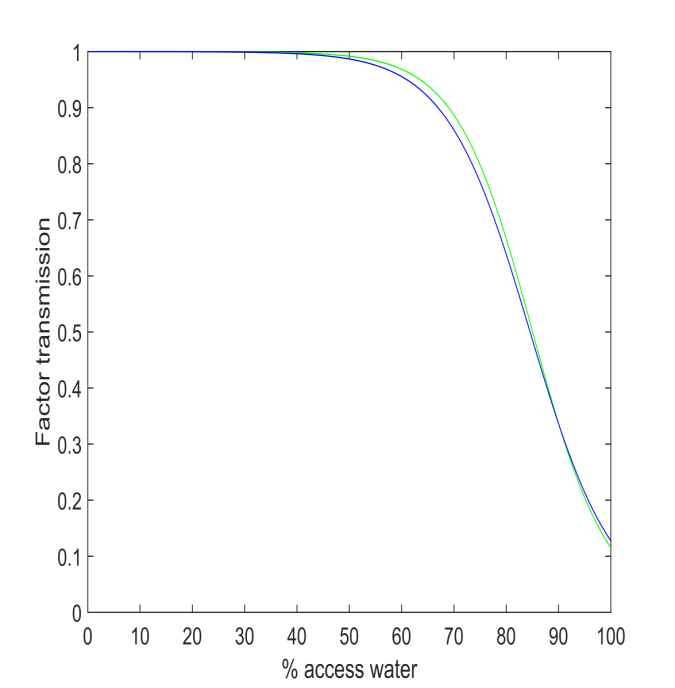 | **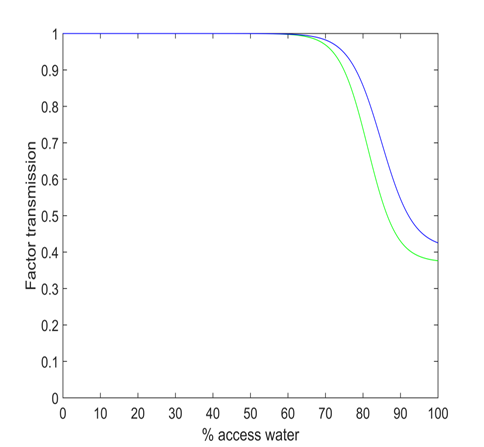** |
| **B** | **D** |
| **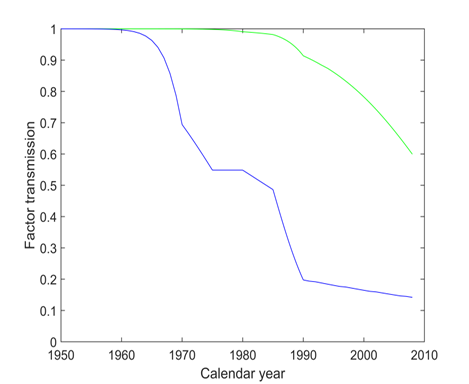** | **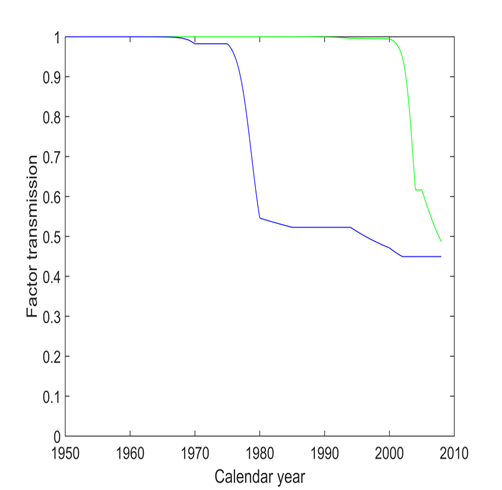** |

Green: rural setting, blue: urban setting

(A) Brazil, Factor for transmission as a function of the percentage with access to safe drinking water

(B) Brazil, Derived factor for transmission as a function of time

(C) Mexico, Factor for transmission as a function of the percentage with access to safe drinking water

(D) Mexico, Derived factor for transmission as a function of time

**G. Sensitivity analyses**

It was assumed in all the analyses that in the base year (1950), 10% of the FOI was by other causes than direct person-to-person transmission. The sensitivity analyses performed and reported here vary the number of parameters of the transmission model that are constrained to have a relative difference between the rural and the urban setting of 10%, so that either three or four parameters are constrained in this way.

**Sensitivity of the model outcomes with respect to the number of parameters constrained**

**Table S4A Brazil**

| **Outcome** | **Setting** | **N pars**  **constr.*** | **1950** | **1975** | **2000** | **2025** | **2050** |
| --- | --- | --- | --- | --- | --- | --- | --- |
| **First age with at least**  **50% HAV+**  **(AMPI)** | **Rural** | **3** | 4 | 5 | 7 | 13 | 14 |
|  |  | **4** | 4 | 4 | 7 | 13 | 14 |
|  | **Urban** | **3** | 2 | 3 | 14 | 20 | 21 |
|  |  | **4** | 3 | 5 | 14 | 18 | 20 |
|  | **Country** | **3** | 3 | 4 | 13 | 19 | 20 |
|  |  | **4** | 3 | 5 | 13 | 18 | 19 |
| **Mean age symptomatic HAV** | **Rural** | **3** | 10 | 10 | 13 | 20 | 25 |
|  |  | **4** | 9 | 9 | 13 | 20 | 24 |
|  | **Urban** | **3** | 5 | 8 | 14 | 23 | 30 |
|  |  | **4** | 8 | 10 | 15 | 24 | 30 |
|  | **Country** | **3** | 9 | 9 | 14 | 23 | 29 |
|  |  | **4** | 9 | 10 | 15 | 23 | 29 |
| **Incidence rate**  **All HAV per year per 100,000** | **Rural** | **3** | 3163 | 2652 | 1477 | 1165 | 983 |
|  |  | **4** | 3254 | 2700 | 1426 | 1168 | 984 |
|  | **Urban** | **3** | 3566 | 2381 | 968 | 1072 | 955 |
|  |  | **4** | 3298 | 2099 | 1136 | 1088 | 954 |
|  | **Country** | **3** | 3309 | 2487 | 1063 | 1083 | 958 |
|  |  | **4** | 3270 | 2334 | 1191 | 1097 | 956 |
| **Incidence rate symptomatic HAV**  **per year per 100,000** | **Rural** | **3** | 695 | 672 | 535 | 622 | 573 |
|  |  | **4** | 608 | 591 | 499 | 619 | 570 |
|  | **Urban** | **3** | 303 | 388 | 416 | 647 | 620 |
|  |  | **4** | 564 | 529 | 526 | 654 | 615 |
|  | **Country** | **3** | 553 | 499 | 439 | 644 | 615 |
|  |  | **4** | 592 | 554 | 521 | 650 | 611 |

* 3 parameters constrained for the base case

**Table S4B Mexico**

| **Outcome** | **Setting** | **N pars**  **constr.*** | **1950** | **1975** | **2000** | **2025** | **2050** |
| --- | --- | --- | --- | --- | --- | --- | --- |
| **First age with at least 50% HAV+**  **(AMPI)** | **Rural** | **3** | 3 | 3 | 4 | 10 | 11 |
|  |  | **4** | 4 | 4 | 5 | 8 | 9 |
|  | **Urban** | **3** | 3 | 3 | 8 | 10 | 12 |
|  |  | **4** | 4 | 5 | 7 | 10 | 12 |
|  | **Country** | **3** | 3 | 3 | 6 | 10 | 12 |
|  |  | **4** | 4 | 4 | 7 | 9 | 12 |
| **Mean age symptomatic HAV** | **Rural** | **3** | 8 | 8 | 9 | 16 | 21 |
|  |  | **4** | 9 | 9 | 11 | 15 | 19 |
|  | **Urban** | **3** | 8 | 8 | 13 | 18 | 22 |
|  |  | **4** | 10 | 10 | 14 | 18 | 22 |
|  | **Country** | **3** | 8 | 8 | 13 | 18 | 22 |
|  |  | **4** | 9 | 10 | 13 | 17 | 21 |
| **Incidence rate**  **All HAV per year**  **per 100,000** | **Rural** | **3** | 3348 | 3292 | 2143 | 1332 | 1027 |
|  |  | **4** | 3237 | 3143 | 2069 | 1351 | 1031 |
|  | **Urban** | **3** | 3346 | 3234 | 1665 | 1305 | 1017 |
|  |  | **4** | 3189 | 2931 | 1902 | 1302 | 1014 |
|  | **Country** | **3** | 3347 | 3256 | 1786 | 1310 | 1019 |
|  |  | **4** | 3217 | 3010 | 1944 | 1311 | 1016 |
| **Incidence rate symptomatic HAV**  **per year per 100,000** | **Rural** | **3** | 513 | 511 | 474 | 618 | 547 |
|  |  | **4** | 619 | 611 | 594 | 562 | 500 |
|  | **Urban** | **3** | 515 | 518 | 603 | 634 | 561 |
|  |  | **4** | 664 | 729 | 714 | 619 | 549 |
|  | **Country** | **3** | 514 | 516 | 571 | 631 | 559 |
|  |  | **4** | 639 | 685 | 684 | 608 | 542 |

* 3 parameters constrained for the base case
